# A Pragmatic Account of the Weak Evidence Effect

**DOI:** 10.1162/opmi_a_00061

**Published:** 2022-09-28

**Authors:** Samuel A. Barnett, Thomas L. Griffiths, Robert D. Hawkins

**Affiliations:** Department of Computer Science, Princeton University, Princeton, New Jersey; Department of Psychology, Princeton University, Princeton, New Jersey

**Keywords:** communication, persuasion, pragmatics, decision-making

## Abstract

Language is not only used to transmit neutral information; we often seek to *persuade* by arguing in favor of a particular view. Persuasion raises a number of challenges for classical accounts of belief updating, as information cannot be taken at face value. How should listeners account for a speaker’s “hidden agenda” when incorporating new information? Here, we extend recent probabilistic models of recursive social reasoning to allow for persuasive goals and show that our model provides a *pragmatic* account for why weakly favorable arguments may backfire, a phenomenon known as the weak evidence effect. Critically, this model predicts a systematic relationship between belief updates and expectations about the information source: weak evidence should only backfire when speakers are expected to act under persuasive goals and prefer the strongest evidence. We introduce a simple experimental paradigm called the *Stick Contest* to measure the extent to which the weak evidence effect depends on speaker expectations, and show that a pragmatic listener model accounts for the empirical data better than alternative models. Our findings suggest further avenues for rational models of social reasoning to illuminate classical decision-making phenomena.

“Well, he would [say that], wouldn’t he?”

—*Mandy Rice-Davies, 1963*

## INTRODUCTION

Communication is a powerful engine of learning, enabling us to efficiently transmit complex information that would be costly to acquire on our own (Henrich, 2015; Tomasello, 2009). While much of what we know is learned from others, it can also be challenging to know how to incorporate socially transmitted information into our beliefs about the world. Each source is a person with a “hidden agenda” encompassing their own beliefs and desires and biases, and not all information can be treated the same (Hovland et al., 1953; O’Keefe, 2015). For example, when deciding whether to buy a car, we may weight information differently depending on whether we heard it from a trusted family memory or the dealership, as we know the dealership is trying to make a sale. While such reasoning is empirically well-established—even young children are able to discount information from untrustworthy or unknowledgeable individuals (Gweon et al., 2014; Harris et al., 2018; Mills & Landrum, 2016; Poulin-Dubois & Brosseau-Liard, 2016; Sobel & Kushnir, 2013; Wood et al., 2013)—these phenomena have continued to pose a problem for formal models of belief updating, which typically take information at face value.

Recent probabilistic models of social reasoning have provided a mathematical framework for understanding how listeners ought to draw inferences from socially transmitted information. Rather than treating information as a direct observation of the true state of the world, social reasoning models suggest treating the true state of the world as a *latent variable* that can be recovered by inverting a generative model of how an intentional agent would share information under different circumstances (Baker et al., 2017; Goodman & Frank, 2016; Goodman & Stuhlmüller, 2013; Hawthorne-Madell & Goodman, 2019; Jara-Ettinger et al., 2016; Vélez & Gweon, 2019; Whalen et al., 2017). These models raise new explanations for classic effects in the judgment and decision-making literature, where behavior is often measured in social or linguistic contexts (Bagassi & Macchi, 2006; Ma et al., 2020; McKenzie & Nelson, 2003; Mosconi & Macchi, 2001; Politzer & Macchi, 2000; Sperber et al., 1995).

Consider the *weak evidence effect* (Fernbach et al., 2011; Lopes, 1987; McKenzie et al., 2002) or *boomerang effect* (Petty, 2018), a striking case of non-monotonic belief updating where weak evidence in favor of a particular conclusion may backfire and actually reduce an individual’s belief in that conclusion. For example, suppose a juror is determining the guilt of a defendant in court. After hearing a prosecutor give a weak argument in support of a guilty verdict—say, calling a single witness with circumstantial evidence—we might expect the juror’s beliefs to only be shifted weakly in support of guilt. Instead, the weak evidence effect describes a situation where the prosecutor’s argument actually leads to a shift in the opposite direction – the juror may now believe that the defendant is more likely to be *innocent*.

Importantly, social reasoning mechanisms are not necessarily in conflict with previously proposed mechanisms for the weak evidence effect, such as algorithmic biases in generating alternative hypotheses (Dasgupta et al., 2017; Fernbach et al., 2011), causal reasoning about other non-social attributes of the situation (Bhui & Gershman, 2020), or sequential belief-updating (McKenzie et al., 2002; Trueblood & Busemeyer, 2011). Both social and asocial models are able to account for the basic effect. To find *unique* predictions that distinguish models with a social component, then, we argue that we must shift focus from the *existence* of the effect to asking *under what conditions* it emerges. Social mechanisms lead to unique predictions about these conditions that purely asocial models cannot generate. In particular, if evidence comes from an intentional agent who is expected to present the strongest possible argument in favor of their case, then weak evidence would imply the absence of stronger evidence (Grice, 1975); otherwise weak evidence may be taken more at face value. Thus, a pragmatic account predicts a systematic relationship between a listener’s social expectations and the strength of the weak evidence effect:^1^ *weak evidence should only backfire when the information source is expected to provide the strongest evidence available to them*.

In this paper, we proceed by first extending recent rational models of communication to equip speakers with persuasive goals (rather than purely informative ones) and present a series of simulations deriving key predictions from our model. We then introduce a simple behavioral paradigm, the *Stick Contest*, which allows us to elicit a participant’s social expectations about the speaker alongside their inferences as listeners. Based on the speaker expectations, we find that participants cluster into sub-populations of *pragmatic* listeners or *literal* listeners, who expect speakers to provide strongly persuasive evidence or informative but neutral evidence, respectively. As predicted by the pragmatic account, only the first group of participants, who expected speakers to provide persuasive evidence, reliably displayed a weak evidence effect in their belief updates. Finally, we use these data to quantitatively compare our model against prior asocial accounts and find that a pragmatic model accounting for these hetereogenous groups is most consistent with the empirical data. Taken together, we suggest that pragmatic reasoning mechanisms are central to explaining belief updating when evidence is presented in social contexts.

## FORMALIZING A PRAGMATIC ACCOUNT OF THE WEAK EVIDENCE EFFECT

To derive precise behavioral predictions, we begin by formalizing the pragmatics of persuasion in a computational model. Specifically, we draw upon recent progress in the Rational Speech Act (RSA) framework (Franke & Jäger, 2016; Goodman & Frank, 2016; Scontras et al., 2018). This framework instantiates a theory of recursive social inference, whereby listeners do not naively update their beliefs to reflect the information they hear, but explicitly account for the fact that speakers are intentional agents choosing which information to provide (Grice, 1975).

### Reasoning about Evidence from Informative Speakers

We begin by defining a pragmatic listener *L* who is attempting to update their beliefs about the underlying state of the world *w* (e.g., the guilt or innocence of the defendant), after hearing an utterance *u* (e.g., an argument provided by the prosecution). According to Bayes’ rule, the listener’s posterior beliefs about the world *P*_*L*_(*w*|*u*) may be derived as follows:

$$P_{L}\left(w|u\right)\propto P_{S}\left(u|w\right)P\left(w\right)$$ (1)

where *P*(*w*) is the listener’s prior beliefs about the world and the likelihood *P*_*S*_(*u*|*w*) is derived by imagining what a hypothetical speaker agent would choose to say in different circumstances. This term yields different predictions given different assumptions about the speaker, captured by different speaker utility functions *U*. In existing RSA models, the speaker is usually assumed to be *epistemically informative*, choosing utterances that bring the listener’s beliefs as close as possible to the true state of the world, as measured by information-theoretic surprisal:

$$\begin{array}{l} P_{S}\left(u|w\right)\propto \exp\left\{\alpha U_{\text{epi}}\left(u;w\right)\right\}\\ U_{\text{epi}}\left(u;w\right)=\ln P_{L_{0}}\left(w|u\right) \end{array}$$ (2)

where the free parameter *α* ∈ [0, ∞] controls the temperature of the soft-max function and *U*_epi_ denotes the utility function of an (epistemically) informative speaker. As *α* → ∞, the speaker increasingly chooses the single utterance with the highest utility, and as *α* → 0 the speaker becomes indifferent among utterances. If this hypothetical speaker, in turn, aimed to be informative to the same listener defined in Equation 1, it would yield an infinite recursion: the RSA framework instead assumes that the recursion is grounded in a base case known as the “literal” listener, *L*_0_, who takes evidence at face value:

$$P_{L_{0}}\left(w|u\right)\propto \delta_{〚u〛\left(w\right)}P\left(w\right).$$ (3)

Here, 〚*u*〛 gives the literal semantics of the utterance *u*, with *δ*_〚*u*〛(*w*)_ returning 1 if *w* is consistent with the state of affairs denoted by *u*, and 0 (or very small *ϵ*) otherwise.

### Reasoning about Evidence from Motivated Speakers

The epistemic utility defined in Equation 2 aims only to produce assertions that most effectively lead to *true* beliefs. Often, however, speakers do not seek to neutrally inform, but to persuade in favor of a particular outcome or “hidden agenda.” What is needed to represent such persuasive goals in the RSA framework? We begin by assuming that motivated speakers have a particular goal state w* that they aim to induce in the listener, where w* does not necessarily coincide with the true state of affairs *w*. This naturally yields a persuasive utility *U*_pers_ that aims to persuade the listener to adopt the intended beliefs w*:

$$U_{\text{pers}}\left(u;w^{*}\right)=\ln P_{L_{0}}\left(w^{*}|u\right)$$ (4)

where we say an utterance *u* is strictly more persuasive than *u*′ if and only if *U*_pers_(*u*|w*) > *U*_pers_(*u*′|w*) (i.e., when the utterance results in the listener assigning higher probability to the desired state w*). Following prior extensions of the speaker utility to other non-epistemic goals (e.g., Bohn et al., 2021; Yoon et al., 2018, 2020), we then define a combined utility assuming the speaker aims to jointly fulfill persuasive aims (Equation 4) while remaining consistent with the true world state *w* (Equation 2):

$$P_{S}\left(u|w,w^{*}\right)\propto \exp\left\{\alpha U\left(u;w,w^{*}\right)\right\}$$ (5)

$$U\left(u;w,w^{*}\right)=U_{\text{epi}}\left(u;w\right)+\beta U_{\text{pers}}\left(u;w^{*}\right)$$ (6)

where *β* is a parameter controlling the strength of the persuasive goal (we recover the standard epistemic RSA model when *β* = 0). This motivated speaker forms the foundation for a pragmatic model of the weak evidence effect.^2^ A pragmatic listener *L*_1_ who suspects that the utterance was generated by a motivated speaker with non-zero bias *β* is able to be “skeptical” of the speaker’s agenda and discount their evidence accordingly:^3^

$$P_{L}\left(w|u,w^{*},\beta \right)\propto P_{S}\left(u|w^{*},w,\beta \right)P\left(w\right)$$ (7)

To see why this model allows evidence to backfire, note that the probability of different utterances are in competition with one another under the speaker model. In the case that *w* and w* coincide, the speaker is expected to choose a utterance that is strongly supportive of that state; weaker utterances have a lower probability of being chosen. Conversely, if w* deviates from the true state of affairs, stronger utterances in favor of w* will be dispreferred (because they will be false and violate the epistemic term), hence weaker utterances are more likely. In this way, the absence of strong evidence from a speaker who would be highly motivated to show it statistically implies that no such evidence exists.

## EXPERIMENT: THE STICK CONTEST

**Figure 1.**
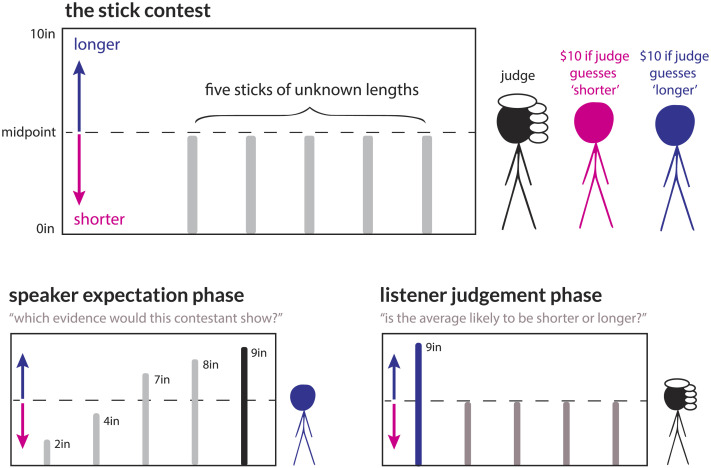
**In the Stick Contest paradigm, participants are asked to determine whether a set of five hidden sticks is longer or shorter, on average, than a midpoint (dotted line) based on limited evidence from a pair of contestants.** In the *speaker expectation* phase (left), participants were asked which one of the five sticks a given contestant would be most likely to show. In the *listener judgment* phase (right), participants were presented with a sequence of sticks from each contestant and asked to judge the likelihood that the overall sample is “longer.”

Empirical studies of the weak evidence effect require a cover story to elicit belief judgments and manipulate the strength of evidence. Typically, this cover story is based on a real-world scenario such as a jury trial (McKenzie et al., 2002) or public policy debate (Fernbach et al., 2011), where participants are asked to report their belief in a hypothetical state such as the defendant’s guilt or the effectiveness of the policy intervention. While these cover stories are naturalistic, they also introduce several complications for evaluating models of belief updating: participants may bring in different baseline expectations based on world knowledge and the absolute scalar argument strength of verbal statements is often unclear. To address these concerns, we introduce a simple behavioral paradigm called *the Stick Contest* (see Figure 1). This game is inspired by a courtroom scenario: two contestants take turns presenting competing evidence to a judge, who must ultimately issue a verdict. Here, however, the verdict concerns the average length of *N* = 5 sticks which range from a minimum length of 1″ to a maximum length of 9″. These sticks are hidden from the judge but visible to both contestants, who are each given an opportunity to reveal exactly one stick as evidence for their case. As in a courtroom, each contestant has a clear agenda that is known to the judge: one contestant is rewarded if the judge determines that the average length of the sticks is longer than the midpoint of 5″ (shown as a dotted line in Figure 1), and the other is rewarded if the judge determines that the average length of the sticks is shorter than the midpoint.

This paradigm has several advantages for comparing models of the weak evidence effect. First, unlike verbal statements of evidence, the scale of evidence strength is made explicit and provided as common knowledge to the judge and contestants. The strength of a given piece of evidence is directly proportional to the length of the revealed stick, and these lengths are bounded between the minimum and maximum values. Second, while previous paradigms have operationalized the weak evidence effect in terms of a sequence of belief updates across multiple pieces of evidence (e.g., where the first piece of evidence sets a baseline for the second piece of evidence), common knowledge about the scale allows the weak evidence effect to emerge from a single piece of evidence. This property helps to disentangle the core mechanisms driving the weak evidence effect from those driving *order effects* (e.g., Trueblood & Busemeyer, 2011).

### Participants

We recruited 804 participants from the Prolific crowd-sourcing platform, 723 of whom successfully completed the task and passed attention checks (see Appendix A). The task took approximately 5 to 7 minutes, and each participant was paid $1.40 for an average hourly rate of $14. We restricted recruitment to the USA, UK, and Canada and balanced recruitment evenly between male and female participants. Participants were not allowed to complete the task on mobile or to complete the experiment more than once.

### Design and Procedure

The experiment proceeded in two phases: first, a *speaker expectation* phase, and second, a *listener judgment* phase (see Figure 1). In the speaker expectation phase, we placed participants in the role of the contestants, gave them an example set of sticks {2, 4, 7, 8, 9} and asked them which ones they believed each contestant would choose to show, in order of priority. In the listener judgment phase, we placed participants in the role of the judge and presented them with a sequence of observations. After each observation, they used a slider to indicate their belief about the verdict on a scale ranging from 0 (“average is definitely shorter than five inches”) to 100 (“average is definitely longer than five inches”). It was stated explicitly that the judge knows that there are exactly five sticks, and that each contestant’s incentives are public knowledge. After each phase, we asked participants to explain their response in a free-response box (see Tables S2–S3 for sample responses).

This within-participant design allowed us to examine individual co-variation between the strength of a participant’s weak evidence effect in the listener judgment phase and their beliefs about the evidence generation process in the speaker expectation phase. Critically, while the set of candidate sticks in the speaker expectation phase was held constant across all participants for consistency, the strength of evidence we presented in the listener judgment phase was manipulated in a between-subjects design. The length of the first piece of evidence was chosen from the set {6, 7, 8, 9} when the long-biased contestant went first, and from the set {4, 3, 2, 1} when the short-biased contestant went first, for a total of 4 possible “strength” conditions (measured as the distance of the observation from the midpoint; we assigned more participants to the more theoretically important “weak evidence” condition, i.e., {4, 6}, to obtain a higher-powered estimate). The order of contestants was counterbalanced across participants and held constant across the speaker and listener phase.^4^ Although it was not the focus of the current study, we also presented a second piece of evidence from the other contestant to capture potential order effects (see Appendix B for preliminary analyses).

## RESULTS

### Behavioral Results

Before quantitatively evaluating our model, we first examine its key qualitative predictions. Do participants exhibit a weak evidence effect in their listener judgments at all, and if so, to what extent is variation in the strength of the effect related to their expectations about the speaker? We focus on each participant’s first judgment, provided after the first piece of evidence in the listener phase. This judgment provides the clearest view of the weak evidence effect, as subsequent judgments may be complicated by order effects. We constructed a linear regression model predicting participants’ continuous slider responses. We included fixed effects of evidence strength as well as expectations from the speaker phase (coded as a categorical variable, expecting strongest evidence vs. expecting weaker evidence), and their interaction, along with a fixed effect of whether the first contestant was “short”-biased or “long”-biased. Because the design was fully between-participant (i.e., each participant only provided a single slider response as judge), no random effects were supported.

**Figure 2.**
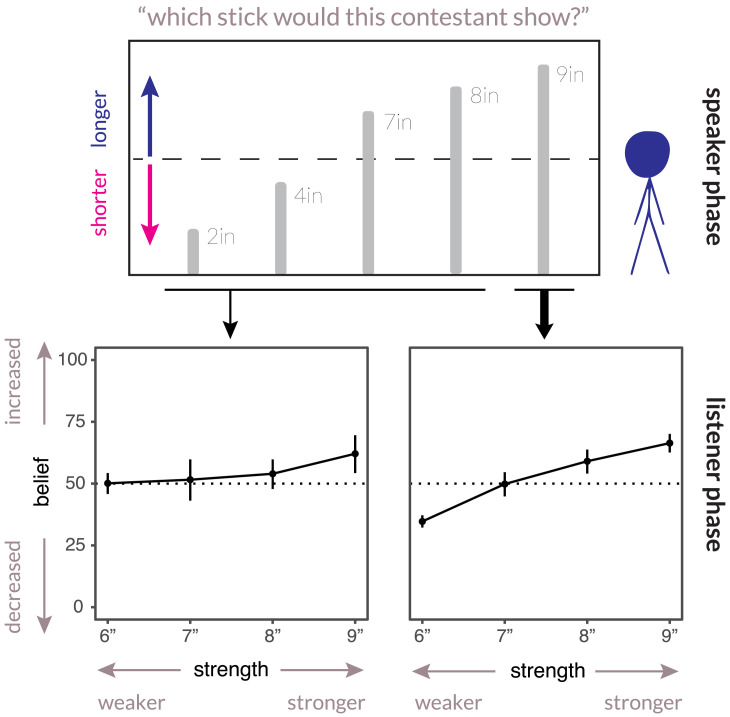
**Individual differences in the weak evidence effect are predicted by pragmatic expectations.** Dotted line represents neutral or unchanged beliefs. Error bars are bootstrapped 95% CIs (see Figure S3 for raw distributions).

As predicted, we found a significant interaction between speaker expectations and evidence strength, *t*(718) = 5.2, *p* < 0.001; see Figure 2. For participants who expected the speaker to provide the strongest evidence (485 participants or 67% of the sample), weak evidence in favor of the persuasive goal backfired and actually pushed beliefs in the opposite direction, *m* = 34.7, 95% CI: [32.3, 37.3], *p* < 0.001. Meanwhile, for participants who expected speakers to “hedge” and not necessarily show the strongest evidence first (238 participants, or 33% of the sample), no weak evidence effect was found (*m* = 50.1, group difference = −15.4, post-hoc *t*(367) = −6.3, *p* < 0.001.) We found only a marginally significant asymmetry in slider bias, *p* = 0.056, with short-biased participants giving slightly larger endorsements (*m* = 1.6 slider points) across the board.

### Model Simulations

The qualitative effect observed the previous section is consistent with our pragmatic account: weak evidence only backfired for participants who expected speakers to provide the strongest available. In this section we conduct a series of simulations to explicitly examine the conditions under which this effect emerges from our model of recursive social reasoning between a speaker (who selects the evidence) and a listener (who updates their beliefs in light of the evidence). Our task is naturally formalized by defining the possible utterances *u* ∈ 𝒰 as the possible lengths of individual sticks the speaker must choose between, the world state *w* as the true set of sticks, and the persuasive goals w* ∈ {longer, shorter} as a binary proposition corresponding to each speaker’s incentive. Because the speaker only has access to true utterances, all utterances have equal epistemic utility (i.e., the speaker must show one of the five *actual* sticks,^5^ which has the epistemic effect of reducing uncertainty about the identity of exactly one stick). Hence, the combined utility (Equation 6) simplifies to the following:

$$S\left(u|w,w^{*},\beta \right)\propto \exp\left\{\alpha \beta \ln\;L_{0}\left(w^{*}|u\right)\right\}$$ (8)

and the persuasive utility of an utterance is monotonic in the stick length (see Appendix C for complete proofs). Note that when *β* = 0, the pragmatic listener *L*_1_ expects the speaker preferences to be uniform over true evidence, *S*_1_(*u* | *w*, w*, *β* = 0) = Unif(*u*), thus reducing to the literal listener *L*_0_. When *β* → ∞, the pragmatic listener expects the speaker to maximize utility and choose the single strongest piece of evidence.^6^

In our simulations, we present the listener models with different pieces of evidence *u* ∈ {5, 6, 7, 8, 9, 10} and manipulate *β*, which represents the degree to which the pragmatic listener *L*_1_ expects the speaker *S* to be motivated to show data that prefers target goal state w* = longer (the case for shorter is analogous). We operationalize the size of the weak evidence effect as the decrease in belief for a proposition given positive evidence supporting that proposition. For example, if observing a stick length of 6″ *decreased* the listener’s beliefs that the sample was longer than 5″ from a prior belief of *P*(longer) = 0.5 to a posterior belief of *P*(longer | *u* = 6) = 0.4, then we say the size of the effect is 0.5 − 0.4 = 0.1.

**Figure 3.**
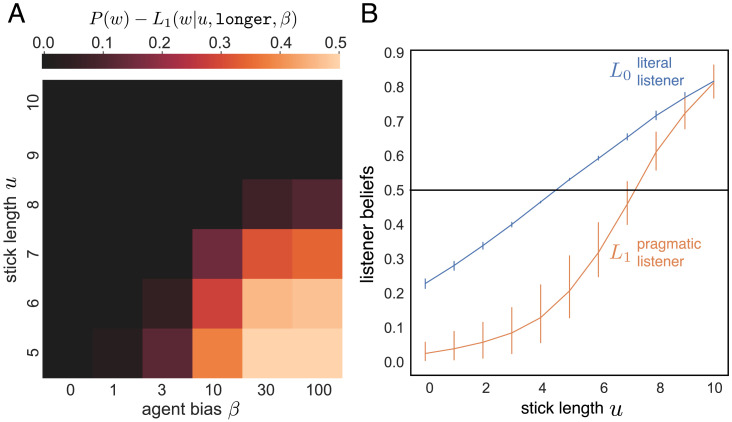
**Model simulations.** (A) Our pragmatic listener model predicts a weak evidence effect for a broader range of evidence strengths at higher perceived speaker bias *β*. The color scale represents the extent to which the listener’s posterior beliefs decrease in light of positive evidence, where the black region represents conditions under which no weak evidence effect is predicted. (B) Posterior beliefs of literal and pragmatic listener models as a function of evidence from long-biased speaker. Horizontal line represents prior beliefs. Error bars are given by 10-fold cross-validation across parameter fits on different subsets of our behavior data, with average $\bar{\beta }$ = 2.03 and response offset $\bar{o}$ = −0.13 (translating the curve down).

First, we observe that when *β* = 0 (Figure 3A, left-most column), no weak evidence effect is observed: the listener interprets the evidence literally. However, as the perceived bias of the speaker increases, we observe a weak evidence effect emerge for shorter sticks. When the perceived bias grows large (e.g., *β* = 100, right-most column), the weak evidence effect is found over a broad range of evidence: if the listener expects the speaker to show the single strongest piece of evidence available, then even a stick of length 8″ rules out the existence of any stronger evidence, shifting the possible range of sticks in the sample. To further understand this effect, we computed the beliefs of literal (*J*_0_) and pragmatic (*J*_1_) listener models as a function of the evidence they’ve been shown (Figure 3B). While the literal listener predicts a near-linear shift in beliefs as a function of positive or negative evidence, the pragmatic listener yields a sharper S-shaped curve reflecting more skeptical belief updating.

### Quantitative Model Comparison

Our behavioral results suggest an important role for speaker expectations in explanations of the weak evidence effect, and our simulations reveal how a pragmatic listener model derives this effect from different expectations about speaker bias. In this section, we compare our model against alternative accounts by fitting them to our empirical data (see Appendix E for details).

#### Fitting the RSA model to behavioral data.

We considered several variants of the RSA model, which handled the relationship between the speaker and listener phase in different ways. The simplest variant, which we call the *homogeneous* model, assumes the entire population of participants is explained by a pragmatic model (*z* = *L*_1_) with an unknown bias. It is homogeneous because the same model is assumed to be shared across the whole population. The second variant, which we call the *heterogeneous* model, is a mixture model where we predicted each participant’s response as a convex combination of the *J*_0_ and *J*_1_ models with mixture weight *p*_*z*_ (i.e., marginalizing out latent assignments *z*_*i*_). In the third variant, which we call the *speaker-dependent* model, we explicitly fit different mixture weights depending on the participant’s response in the speaker expectations phase. Rather than learning a single mixture weight for the entire population, this variant learns independent mixture weights for different sub-groups *z*_*j*_, defined by the different sticks *j* that participants chose in the speaker phase. This model asks whether conditioning on speaker data allows the model to make sufficiently better predictions about the listener data.

#### Fitting anchor-and-adjust models to empirical data.

The most prominent family of *asocial* models accounting for the weak evidence effect are *anchor-and-adjust* (AA) models. In these models, individuals compare the strength of new evidence *u* against a reference point *R* and adjust their beliefs *P*(*w*|*u*) up or down accordingly:

$$P\left(w|u\right)=P\left(w\right)+\eta \left(s\left(u\right)-R\right),$$ (9)

where *s*(*u*) is the strength of the evidence, and *η* is an adjustment weight. In the simplest variant (Hogarth & Einhorn, 1992), the reference point and scaling are fixed to a neutral baseline *η* = *P*(*w*) = 1 − *P*(*w*) = .5 and *R* = 0. In a more complex variant, beliefs are not updated from a *neutral* baseline but instead relative to more stringent level known as the argument’s “minimum acceptable strength” (MAS; McKenzie et al., 2002), which is treated as a free parameter: *R* ∼ Unif[−1, 1]. In this case, positive evidence that falls short of *R* may nonetheless be treated as negative evidence and decrease the listener’s beliefs. Although the anchor is typically taken to be a specific earlier observation, it may be interpreted in the single-observation case as the participant’s implicit or imagined expectations from the task instructions and cover story. Prior work using anchor-and-adjust models would not predict a relationship between behavior in the speaker phase and in the listener phase. We thus evaluated a homogeneous *AA* model, a homogeneous *MAS* model, and a heterogeneous mixture model predicting responses as a convention combination of the two.

#### Comparison results.

We examined several metrics to assess the relative performance of these models.^7^ First, as an absolute goodness of fit measure, we found the parameters that maximized the model likelihood (see Table 1). As a Bayesian alternative, which penalizes models for added complexity, we also considered a measure using the full posterior,^8^ the Watanabe-Akaike (or Widely Applicable) Information Criterion (Gelman et al., 2013; Watanabe, 2013). The WAIC penalizes model flexibility in a way that asymptotically equates to Bayesian leave-one-out (LOO) cross-validation (Acerbi et al., 2018; Gelman et al., 2013), which we also include in the form of the PSIS-LOO measure (PSIS stands for Pareto Smoothed Importance Sampling, a method for stabilizing estimates Vehtari et al., 2017). These comparison criteria (Table 1) suggest that the added complexity of the speaker-dependent RSA model is justified: it outperforms all *asocial* variants. For this speaker-dependent model, we found a maximum *a posteriori* (MAP) estimate of $\hat{\beta }$ = 2.26, providing strong support for a non-zero persuasive bias term. We found that the pragmatic *J*_1_ model best explained the judgments of participants who expected the strongest evidence to be shown during the speaker phase (mixture weight $\hat{p}$_*z*_ = 0.99) while the literal *J*_0_ model best explained the judgments of participants who expected weaker sticks to be shown (mixture weight $\hat{p}$_*z*_ = 0.1). Full parameter posteriors are shown in Figure S5.

**Table 1.** Results of the model comparison, including the likelihood achieved by the best-fitting model as well as the WAIC, and PSIS-LOO (± standard error), which penalize for model complexity.

Model	Variant	Likelihood	WAIC	PSIS-LOO
A&A	Homogeneous	−28.1	57.7 ± 9.9	28.8 ± 9.9
MAS	Homogeneous	8.2	−13.3 ± 9.6	−6.6 ± 9.6
	Heterogeneous	8.2	−11.3 ± 9.5	−5.6 ± 9.5
RSA	Homogeneous	8.1	−13.3 ± 9.5	−6.7 ± 9.5
	Heterogeneous	8.1	−10.5 ± 9.3	−5.2 ± 9.3
	Speaker-dependent	**12.0**	**−16.4** ± 9.1	**−9.2** ± 9.1

## DISCUSSION

Evidence is not a direct reflection of the world: it comes from somewhere, often from other people. Yet appropriately accounting for social sources of information has posed a challenge for models of belief-updating, even as increasing attention has been given to the role of pragmatic reasoning in classic phenomena. In this paper, we formalized a pragmatic account of the *weak evidence effect* via a model of recursive social reasoning, where weaker evidence may backfire when the speaker is expected to have a persuasive agenda. This model critically predicts that individual differences in the weak evidence effect should be related to individual differences in how the speaker is expected to select evidence. We evaluated this qualitative prediction using a novel behavioral paradigm—the Stick Contest—and demonstrated through simulations and quantitative model comparisons that our model uniquely captures this source of variance in judgments.

Several avenues remain important for future work. First, while we focused on the initial judgment as the purest manifestation of the weak evidence effect, subsequent judgments are consistent with the *order effects* that have been the central focus of previous accounts (see Appendix B; Anderson, 1981; Davis, 1984; Trueblood & Busemeyer, 2011). Thus, we view our model of social reasoning as capturing an orthogonal aspect of the phenomenon, and further work should explicitly integrate computational-level principles of social reasoning with process-level mechanisms of sequential belief updating. Second, our model provides a foundation for accounting for related *message involvement* effects (e.g., emotion, attractiveness of source), *presentation* effects (e.g., numerical vs. verbal descriptions), and *social affiliation* effects (i.e., whether the source is in-group) that have been examined in real-world settings of persuasion (e.g., Bohner et al., 2002; Cialdini, 1993; DeBono & Harnish, 1988; Falk & Scholz, 2018; Martire et al., 2014; Park et al., 2007), These settings also involve uncertainty about the *scale* of possible argument strength, unlike the clearly defined interval of lengths in our paradigm. Third, while the weak evidence effect emerges after a single level of social recursion, it is natural to ask what happens at higher levels: what about a more sophisticated speaker who is *aware* that weak evidence may lead to such inferences? Our paradigm explicitly informed participants of the speaker bias, but uncertainty about the speaker’s hidden agenda may give rise to a *strong* evidence effect (Perfors et al., 2018), where speakers are motivated to *avoid* the strongest arguments to appear more neutral (see Appendix E). Based on the self-explanations we elicited (Table S2), it is possible that some participants who expected less strong evidence were reasoning in this way. These individual differences are consistent with prior work reporting heterogeneity in levels of reasoning in other communicative tasks (e.g., Franke & Degen, 2016).

We used a within-participant individual differences design for simplicity and naturalism, but there are also limitations associated with this design choice. For example, it is possible that the group of participants who expected weaker evidence to be shown first could be systematically different from the other group in some way, such as differing levels of inattention or motivation, that explains their behavior on *both* speaker and listener trials. We aimed to control for these factors in multiple ways, including strict attention checks (Appendix A) and self-explanations (Tables S2–S3), which suggest a thoughtful rationale for expecting weaker evidence. However, an alternative solution would be to explicitly manipulate social expectations about the speaker in the cover story (e.g., training participants on speakers that tend to show weaker or stronger evidence first). Such a design would license stronger causal inferences, but would also raise new concerns about exactly what is being manipulated. A second limitation of our design is that the speaker phase was always presented before the listener phase. It is already known that the order of these roles may affect participants’ reasoning (e.g., Shafto et al., 2014; Sikos et al., 2021), but asocial accounts of the weak evidence effect would not predict any relationship between speaker and listener trials under *either* order. Hence, we chose the order we thought would minimize confusion about the task; it is not our goal to suggest that social reasoning is spontaneous or mandatory, and we expect that social-pragmatic factors may be more salient in some contexts than others (e.g., when evidence is presented verbally vs. numerically, as in Martire et al., 2014).

Probabilistic models have continually emphasized the importance of the data generating process, distinguishing between assumptions like *weak* sampling, *strong* sampling, and *pedagogical* sampling (Hsu & Griffiths, 2009; Shafto et al., 2014; Tenenbaum, 1999; Tenenbaum & Griffiths, 2001). Our work considers a fourth sampling assumption, *rhetorical sampling*, where the data are not necessarily generated in the service of pedagogy but rather in the service of persuasive rhetoric. Critically, although we formalized this account in a recursive Bayesian reasoning framework, insights about rhetorical sampling are also compatible with other frameworks: for example, work in the anchor-and-adjust framework may use similar principles to derive a relationship between information sources and reference points. Such socially sensitive objectives may be particularly key in the context of developing artificial agents that are more closely aligned with human values (Carroll et al., 2019; Hilgard et al., 2021; Irving et al., 2018). As we navigate an information landscape increasingly filled with disinformation from adversarial sources, a heightened sense of skepticism may be rational after all.

## ACKNOWLEDGMENTS

This work was supported by grant #62220 from the John Templeton Foundation to TG. RDH is funded by a C.V. Starr Postdoctoral Fellowship and NSF SPRF award #1911835. We are grateful for early contributions by Mark Ho and helpful conversations with other members of the Princeton Computational Cognitive Science Lab, as well as Ryan Adams and members of the Laboratory for Intelligent Probabilistic Systems.

## Notes

^1^ Harris et al. (2013) presents a related model of the *faint praise* effect, where the omission of any stronger information that a speaker would be expected to know implies that it is more likely to be negative than positive (e.g., “James has very good handwriting.”) Importantly, this effect is sensitive to the perceived expertise of the source; no such implication follows for unknowledgable informants (see also Bonawitz et al., 2011; Gweon et al., 2014; Hsu et al., 2017, for related inferences from omission).^2^ Coincident with our work, Vignero (2022) has proposed a similar formulation to explain how speakers may stretch the truth of epistemic modals like “possibly” or “probably.”^3^ Although we formulate the listener’s posterior as being conditioned on a *known* value of *β*, we can also consider the case in which the listener has a prior distribution over biases and can compute (marginal) posteriors accordingly—refer to Appendix E for details.^4^ An earlier iteration of our experiment only used a long-biased speaker; we report results from this version in Appendix D.^5^ For related tasks studying outright lying, see Franke et al. (2020), Oey et al. (2019), Oey and Vul (2021), and Ransom et al. (2017). For a more comprehensive and multidisciplinary overview of varieties of deception and misleading, see Meibauer (2019) and Saul (2012).^6^ Because the product *α* · *β* is non-zero only if the persuasion weight *β* is non-zero, these two parameters are redundant in our task. We thus treat their product as a single free parameter, effectively fixing *α* = 1. It is possible that a near-zero *α* (e.g., low effort from participants) may make it difficult to empirically detect a non-zero *β* term in our model comparison below, but this would work against our hypothesis.^7^ All models were implemented in WebPPL (Goodman & Stuhlmüller, 2014); code for reproducing these analyses is available at https://github.com/s-a-barnett/bayesian-persuasion.^8^ We drew 1,000 samples from the posterior via MCMC across four chains, with a burn-in of 7,500 steps and a lag of 100 steps between samples.
