# Supplementary material for: A Pragmatic Account of the Weak Evidence Effect
Source: Open Mind (Camb). 2022 Sep 28;6:169–82. doi: 10.1162/opmi_a_00061 (PMC9692057; doi:10.1162/opmi_a_00061)
Supplement: Supplementary file 1 [file opmi-06-169-s001.pdf]

Journal: **OPEN MIND**

Appendix for "A pragmatic account of the weak evidence effect"

Samuel A. Barnett, Thomas L. Griffiths and Robert D. Hawkins

APPENDIX A: EXCLUSIONS AND ATTENTION CHECKS

Our pre-registered exclusion criteria used two basic attention checks. First, participants were required to complete a comprehension quiz immediately following the task instructions, and we excluded participants who failed to successfully complete this quiz within three attempts. Second, at the end of the experiment, we asked participants to use a slider to indicate the degree of bias they believed each contestant exhibited. These motivations were stated explicitly in the instructions (e.g. "the red contestant will receive $10 if the judge chooses "shorter," otherwise the blue contestant will receive $10") so, although participants may differ in the *degree* to which they thought such incentives would bias the contestants away from neutrality, we took responses in the *opposite* direction of the incentive as indicative of inattentiveness or misunderstanding of task instructions.

We therefore coded bias check responses as "incorrect" if the slider response was inconsistent with the bias given in the instructions (e.g. if the short-biased contestant received a slider rating above the midpoint, $s \geq 50 - \epsilon$, or the long-biased contestant received a slider rating below the midpoint, $s \leq 50 + \epsilon$ where we set $\epsilon = 5$ to allow for the possibility of motor jitter from participants who intended to use the exact midpoint.) In our pre-registered second sample (reported in the main text), 793 participants completed instructions and 723 (91%) of them passed the attention check.

While these pre-registered criteria were designed to ensure that apparent differences in speaker and listener behavior were not simply driven by general attentional factors, it is possible that participants who did not expect the strongest evidence to be shown in the speaker phase (238 participants, or 33%) were still systematically less attentive than other participants. To address this concern, we analyzed a series of other measures to assess the degree of attention and task understanding across "speaker expectation" groups. Specifically, we examine internal consistency within several post-test questions, where we asked participants (i) to make a final two-alternative forced choice verdict about whether the sample of sticks is 'longer' vs. 'shorter' than 5 inches, (ii) to provide a point estimate of their best guess of the actual mean on a slider ranging from 1 inch to 9 inches, and (iii) to guess the values of the remaining three sticks that

Copyright ©2022 The Author(s). Published by MIT Press. All Rights Reserved.

group	n	2AFC + point estimate consistent	generative model also consistent
strongest first	485	0.97	0.89
not strongest first	238	0.96	0.86

Table S1: Stricter attention check passage rates broken out by speaker group.

were not revealed, allowing us to impute a "generative" average across the two observed values and the three guessed values (Table S1).

We say a participant passed the 2AFC check if their binary verdict ('longer' vs. 'shorter') is consistent with the direction of their point estimate. We say a participant also passed the stricter "generative" check if the average imputed from their guesses for the remaining three unobserved sticks matches their 2AFC and point estimates. We observe that rates for the these stricter checks were somewhat lower for participants who expected speakers not to show the strongest evidence first (97% vs. 96%, and 89% vs. 86%, respectively), though neither of these differences was significant, $\chi^2(1) = 0.76, p = 0.38$ and $\chi^2(1) = 1.05, p = 0.31$, respectively. Rates were far above chance for all groups. To ensure robustness, we re-ran our primary analyses on the subset of participants that passed the strictest conjunction of all checks, which is highly improbable under an inattentive null model, and obtained nearly identical results (most crucially, a significant interaction, $t(718) = 5.18, p < 0.001$).

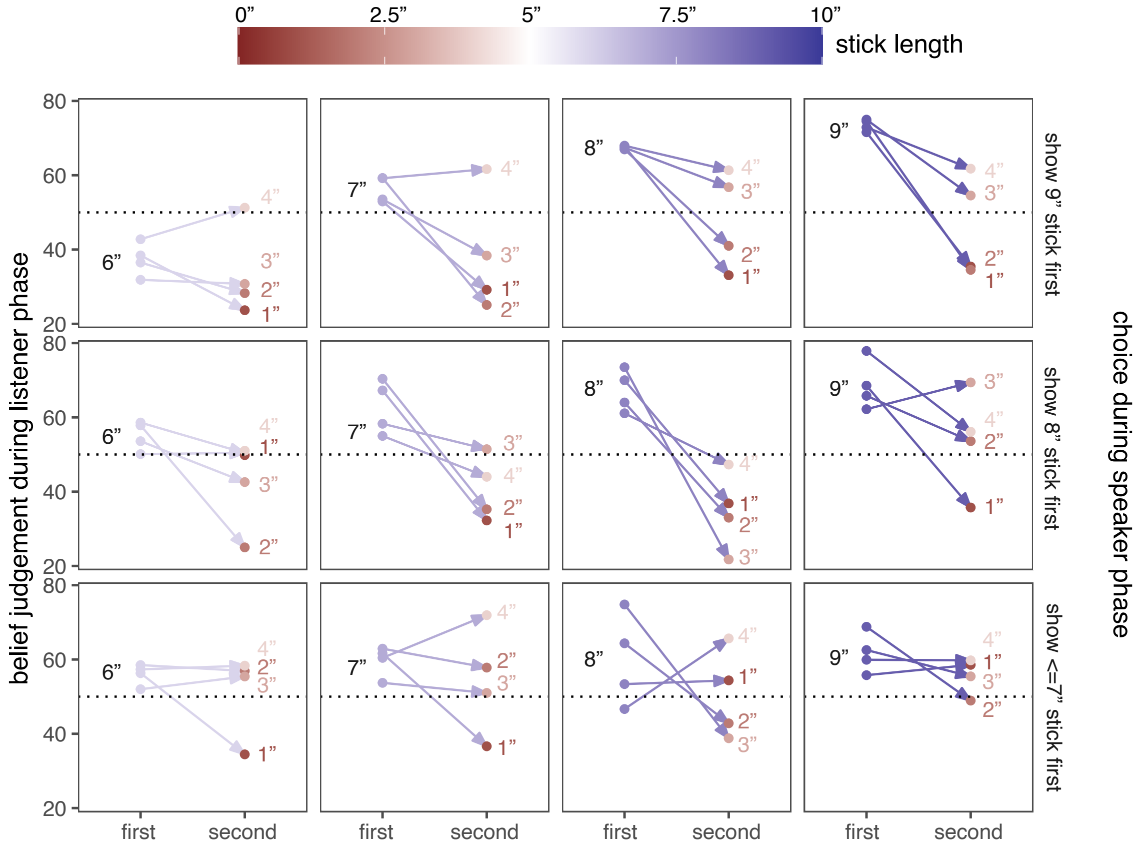

Figure S1: Participants revised their beliefs after obtaining a second piece of evidence. Each facet represents participants who were given the same initial piece of evidence (blue dots) with each arrow connecting their judgment after the first piece of evidence and the second piece of evidence. In most cases, participants revised their estimates down, although participants who showed a weak evidence effect for the first stick (top column) also displayed a classical weak evidence effect on the second piece of evidence (e.g. in the second row, participants who saw a 7" stick on the first trial were slightly *more* confident the average was longer after seeing a 4" stick).

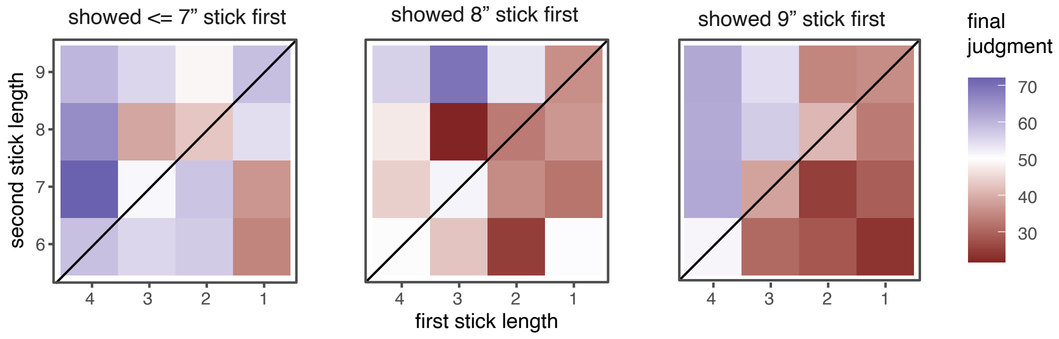

Figure S2: We found strong order effects, with the belief judgment elicited after the second stick apparently affected by a recency bias. Under perfect averaging, the diagonal would leave the judge with complete uncertainty (denoted on our color scale by white), since the evidence from both the longer side (blue) and the shorter side (red) should cancel out.

APPENDIX B: ORDER EFFECTS

While we focus on the first piece of evidence as the clearest weak evidence effect, we also collected a second response after a second piece of evidence was shown by the other speaker. These responses are visualized in Fig. S1. As expected, we observed a recency effect (more easily observed in the diagonal of Fig. S2, where evidence from the "short"-biased and "long"-biased speakers were equally strong), where participants weighted the second piece of evidence more strongly.

APPENDIX C: PROOFS

Theorem 1. *The speaker model using the combined utility Eq. 6 simplifies to Eq. 8 for the stick contest task.*

Proof. We begin by substituting the combined utility (Eq. 6) into the speaker softmax:

$$
\begin{aligned}
S(u|w, w^*) &\propto \exp\{\alpha \cdot U(u; w, w^*)\} \\
&= \exp\{\alpha \cdot [U_{\text{epi}}(u; w) + \beta \cdot U_{\text{pers}}(u; w^*)]\} \\
&= \exp\{\alpha \cdot U_{\text{epi}}(u; w)\} \cdot \exp\{\alpha \cdot \beta \cdot U_{\text{pers}}(u; w^*)\}
\end{aligned}
$$

Now, using Eq. 3 to expand the first term, note that

$$U_{\text{epi}}(u; w) = \ln P_{L_0}(w|u) = \ln \frac{P(w)\delta_{[\![u]\!](w)}}{\sum_w P(w)\delta_{[\![u]\!](w)}} = \begin{cases} -\ln N & \text{if } [\![u]\!](w) \\ -\infty & \text{o.w.} \end{cases}$$

where N is the number of sticks in the true set ($N = 5$ in our experiment). However, we already assume that the set of possible utterances $\mathcal{U}$ are the true sticks in the underlying set (i.e. the contestants cannot make up sticks, they must choose one of the N sticks in the set), so

$$\begin{aligned} \exp\{\alpha \cdot U_{\text{epi}}(u, w)\} &= \begin{cases} \alpha/N & \text{if } [\![u]\!](w) \\ 0 & \text{o.w.} \end{cases} \\ &= \alpha/N \end{aligned}$$

Because all utterances have the exact same epistemic utility U_{epi}, this term drops out of the soft-max:

$$\begin{aligned} S(u|w, w^*) &\propto \exp\{\alpha \cdot U_{\text{epi}}(u; w)\} \cdot \exp\{\alpha \cdot \beta \cdot U_{\text{pers}}(u; w^*)\} \\ &\propto \exp\{\alpha \cdot \beta \cdot U_{\text{pers}}(u; w^*)\} \\ &= \exp\{\alpha \cdot \beta \cdot \ln L_0(w^*|u)\} \end{aligned}$$

yielding Eq. 8. □

Theorem 2. *Persuasiveness monotonically increases as a function of stick length.*

Proof. We say an utterance u is more persuasive than an utterance u' when

$$U_{\text{pers}}(u \mid w^*) > U_{\text{pers}}(u' \mid w^*).$$

Under the stick contest, let $\mathcal{L} = \{l_1, \ldots, l_N\}$ be an partially-ordered set of N stick lengths, such that $l_i \leq l_j$ for any index $i < j$. We denote the mean stick length by $\bar{l} = \frac{1}{N}\sum_i l_i$. Without loss of generality, let the speaker's persuasive goal be $w^* = \texttt{shorter} = \bar{l} < 5$ (the argument follows analogously for `longer`). Take two utterances $u = l_i$ and $u' = l_j$ such that $l_i \leq l_j$ (i.e. such that u is just as short or

shorter than u'). First, we expand the utility:

$$\begin{aligned} U_{\text{pers}}(u \mid \texttt{shorter}) &= \ln L_0(\texttt{shorter} \mid u) \\ &= \ln P(\bar{l} < 5 \mid l_i) \\ &= \ln P\left(\frac{l_i + \sum l_{-i}}{N} < 5\right) \\ &= \ln P\left(\sum l_{-i} < 5N - l_i\right) \end{aligned}$$

Now, let X be a random variable representing the sum of the $N-1$ still-unknown sticks, $X = \sum l_{-i}$. Then we recognize this as the cumulative distribution function (CDF), $F_X(x) = P(X < x)$. Because the underlying set of sticks $\mathcal{L}$ is assumed to be i.i.d., note that the random variable $X = \sum l_{-i}$ does not depend on the original choice of i. Critically, we know that the cumulative distribution function is monotonic increasing in x, i.e. $F_X(a) \leq F_X(b)$ for $a \leq b$. Hence if $l_i \leq l_j$ then $5N - l_i \geq 5N - l_j$ and $F_X(5N - l_i) \geq F_X(5N - l_j)$:

$$\begin{aligned} U(u \mid \texttt{shorter}) &= \ln P\left(\sum l_{-i} < 5N - l_i\right) \\ &= \ln F_X(5N - l_i) \\ &\geq \ln F_X(5N - l_j) \\ &= U(u' \mid \texttt{shorter}) \end{aligned}$$

□

APPENDIX D: RESULTS FROM ORIGINAL SAMPLE

The results reported in the main text are based on a pre-registered replication we conducted during the revision of the manuscript (May 2022). In this appendix, we report the corresponding results from our original sample (February 2020). The only methodological difference between the original study and the internal replication was the way we counter-balanced the order of the “long”- vs. “short”-biased contestants. In our original study, the “long”-biased contestant always presented their evidence first; in our replication, the order of the contestants was randomized. Additionally, in our replication, we added the following clarification to the instructions: “Sticks ranging in length from 1 to 9 inches are equally likely to appear in the set.” Participants in the initial sample were recruited on the Prolific platform, with

no restriction on country. Of the 784 participants who successfully completed the instructions, 708 passed the second attention check.

Our regression model was the same as in the main text, except we did not include a fixed effect of "long" vs. "short": all participants were shown evidence from the "long"-biased speaker. As in the study reported in the main text, we found a significant interaction between speaker expectations and evidence strength on beliefs about the underlying mean, $t(704) = 5.9, p < 0.001$. For participants who expected the speaker to provide the strongest evidence (421 participants or 60% of our sample), the weak evidence provided by a six inch stick backfired, leading them to instead expect that the mean stick length was significantly less likely to be longer than five inches, $m = 37.5$, 95% CI: $[33.1, 41.9]$, $t(98) = -5.7$, $p < 0.001$. Meanwhile, for participants who expected to be shown the second-longest stick (40% of the sample), no weak evidence effect was found, with the 'longest stick' group significantly different from the other groups, $t(167) = -5.5, p < 0.001$.

APPENDIX E: MODEL FITTING DETAILS

RSA model

We used the following priors for our Bayesian data analysis:

$$\begin{aligned} y &\sim \text{Gaussian}(\mu + o, 0.3) \\ p_z &\sim \text{Unif}[0, 1] \\ \beta &\sim \text{Unif}[0, 10] \\ o &\sim \text{Unif}[-0.5, 0.5] \end{aligned}$$

where p_z is the mixture weight used for heterogeneous models, $\mu = P_{J_i}(\texttt{longer}|u) \in [0, 1]$ is the RSA listener model's posterior belief, and o is a uniform offset included to allow for systematic response biases in use of the slider. Intuitively, Gaussian$(\mu + o, 0.3)$ can be viewed as a simple way of scoring the error between the model prediction $\mu + o$ and the participant's response y. For the speaker-dependent model, we used independent priors depending on the participant's choice of stick j: $p_z^{(j)} \sim \text{Unif}[0, 1]$. Because there were relatively fewer participants who expected the `longer` speaker to choose 0.2 or 0.4 (sticks that were in the opposite direction of their goal; and vice versa for the `shorter` speaker), we collapsed these participants together, forming three groups: those who expected the strongest evidence to

be presented first (e.g. who selected $\{0.2, 0.9\}$ for the *short* and *long* biased speakers, respectively), those who expected the second-strongest to be presented first (e.g. who selected $\{0.4, 0.8\}$, respectively), and those who expected less strong evidence. However, our findings are robust to whether we collapse these groups or not.

Belief-adjustment models

In the notation of McKenzie, Lee, and Chen (2002), Eq. 9 is written:

$$C_k = C_{k-1} + w_k \cdot (s(e_k) - R), \tag{S1}$$

where $C_k \in [0, 1]$ is the degree of belief in a particular claim after being presented with evidence e_k, $s(e_k)$ is the *independently judged* strength of evidence e_k, R is a reference point, and $w_k \in [0, 1]$ is an adjustment weight for evidence e_k. In the *adding* variant of the belief-adjustment model, Hogarth and Einhorn (1992) argue that the evidence should be encoded in an absolute manner, letting $R = 0$ and $s(e_k) \in [-1, 1]$, and assuming that if $s(e_k) \leq R$ then $w_k = C_{k-1}$, otherwise $w_k = 1 - C_{k-1}$.[1] To allow the reference point for evidence to be more demanding than neutrality, McKenzie et al. (2002) proposed replacing the reference point R with a Minimum Acceptable Strength (MAS) threshold $(m \mid e)$, that depends on the evidence previously presented. We can therefore rewrite Eq. S1 as

$$C_k = C_{k-1} + w_k \cdot (s(e_k) - (m_k \mid e_1, ..., e_{k-1})). \tag{S2}$$

To fit this class of models to our data, we follow Trueblood and Busemeyer (2011), assuming a mapping between stick length and evidence strength given by a centered logistic function:

$$\text{strength}(u) = \frac{1}{1 + \exp\left(-B \cdot (u - 5)\right)} - 0.5, \tag{S3}$$

where the logistic growth rate B is fit to the data (we used a uniform prior $B \sim \text{Unif}[0, 10]$). This function satisfies several desiderata: it is monotonically increasing in the size of the stick, it is bounded in

[1] The *averaging* variant, in which evidence is encoded in relationship to the current belief in the hypothesis, is more suited for *estimation* tasks involving some kind of moving average (Hogarth & Einhorn, 1992), whereas the Stick Contest is better described as an *evaluation* task in which a single hypothesis is under consideration (“is the sample long?”). We also found empirically that the adding variant provided a better fit to the data than the averaging variant.

the interval $[-1, 1]$, and it is centered in line with the prior over stick lengths, so that a stick of length 5 inches has a strength of 0.5.

For the anchor-and-adjust (AA) variant, we fix the reference point as $R = 0$, and for the minimum acceptable strength (MAS) variant, we infer a reference point with prior $R \sim \text{Unif}[-1, 1]$. We consider *homogeneous* variants in which the entire population is assumed to share the same model with the same parameters, as well as a *heterogeneous* model, in which we assume *a priori* that participants are a convex combination of the two models. As in the RSA models, we infer the mixture weight p_z that best explains the population-level mixture (marginalizing over latent variable assignments z).

Higher levels of reasoning and the strong evidence effect

While our cover story explicitly provided participants with the motivations of speakers, in terms of their financial incentives, these motivations are less obvious in most real-world scenarios. They must be *inferred* from what the speaker is saying. This is straightforwardly derived in our framework by allowing the listener to jointly infer the true state of the world w *and* the speaker's bias β:

$$P_{L_1}(w, \beta \mid u) \propto P_{S_1}(u \mid w, \beta) \cdot P(w) \tag{S4}$$

Our formulation raises a natural question about how speakers would behave if they were *aware* judges were making such inferences. This emerges at the next level of recursive reasoning:

$$P_{S_2}(u \mid w, \beta) \propto \exp\left(|\beta| \ln\big(P_{L_1}(w^* \mid u) - w_c \cdot C(u)\big)\right). \tag{S5}$$

where $C(u)$ represents some cost associated with being perceived as biased by the judge:

$$C(u) = \mathbb{E}_{\beta \sim P_{L_1}(\cdot|u)}\big[|\beta|\big], \tag{S6}$$

and $w_c \geq 0$ is a parameter specifying the degree of the cost. We included a J_2 model who reasons about this listener in our model comparison (i.e. allowing participants to be explained by a convex combination of all three levels) and found that this three-level speaker-dependent model leads to improved performance over the two-level speaker-dependent model (max likelihood $= 16.2$, WAIC$= -18.3 \pm 8.9$, PSIS-LOO$= -9.2 \pm 8.9$.) We conjecture that this formulation is required to account for the *strong evidence effect* (Perfors, Navarro, & Shafto, 2018), in which the desire to appear unbiased leads a speaker

to choose weaker evidence in spite of the presence of stronger alternatives, but leave further investigation for future work.

APPENDIX F: TRANSCRIPT OF THE EXPERIMENT

The written instructions for our experiment are reproduced below. Note that the task can be seen exactly as participants experienced it (e.g. with images) using the code released in our repository: https://github.com/s-a-barnett/bayesian-persuasion.

> In this task, you will serve as the judge for a heated game between these two contestants. The two contestants in this game have been given a set of sticks ranging in length from very long ones to very short ones. Sticks ranging in length from 1 to 9 inches are equally likely to appear in the set. One contestant (shown in pink) will be rewarded handsomely if they can convince you that the average length of these sticks is shorter than 5in (see dotted line). The other (shown in blue) will get paid if if they can convince you that the average length of these sticks is longer than 5in (see dotted line). In this case, the average length is 6in, so the position that this person was arguing for was true. As the judge, however, you will not be able to see the full set of sticks: you will only see what the contestants choose to show you. They will each get to show exactly one of the five sticks to convince you. After you see each stick, you will use this slider to report how strongly you are leaning in your decision. If you think the stick average is more likely to be shorter than 5in, click further to the left. If you think it is more likely to be longer than 5in, click further to the right.

REFERENCES

Hogarth, R. M., & Einhorn, H. J. (1992). Order effects in belief updating: The belief-adjustment model. *Cognitive Psychology*, *24*(1), 1–55.

McKenzie, C. R. M., Lee, S. M., & Chen, K. K. (2002). When negative evidence increases confidence: Change in belief after hearing two sides of a dispute. *Journal of Behavioral Decision Making*, *15*(1), 1–18.

Perfors, A., Navarro, D., & Shafto, P. (2018). Stronger evidence isn’t always better: The role of social inference in evidence selection. In *Proceedings of the 40th Annual Conference of the Cognitive Science Society* (pp. 864–869).

Trueblood, J. S., & Busemeyer, J. R. (2011). A quantum probability account of order effects in inference. *Cognitive Science*, *35*(8), 1518–1552.

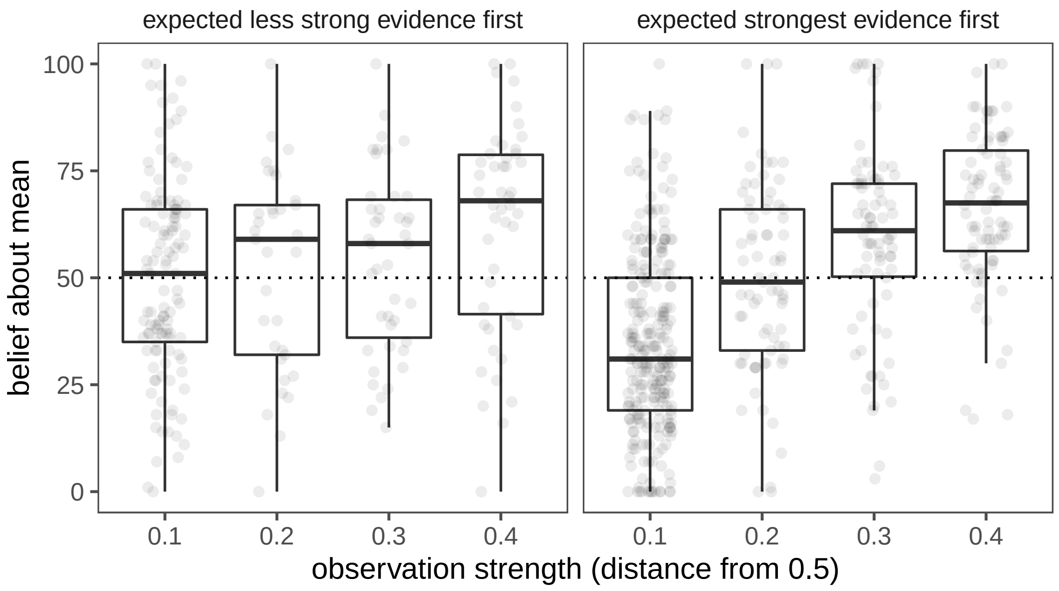

Figure S3: The raw data distribution of responses for the listener phase, where each individual (jittered) point is a different participant and the boxplot represents the median (dark line) and first and third quartiles (top and bottom of box) of the response distribution.

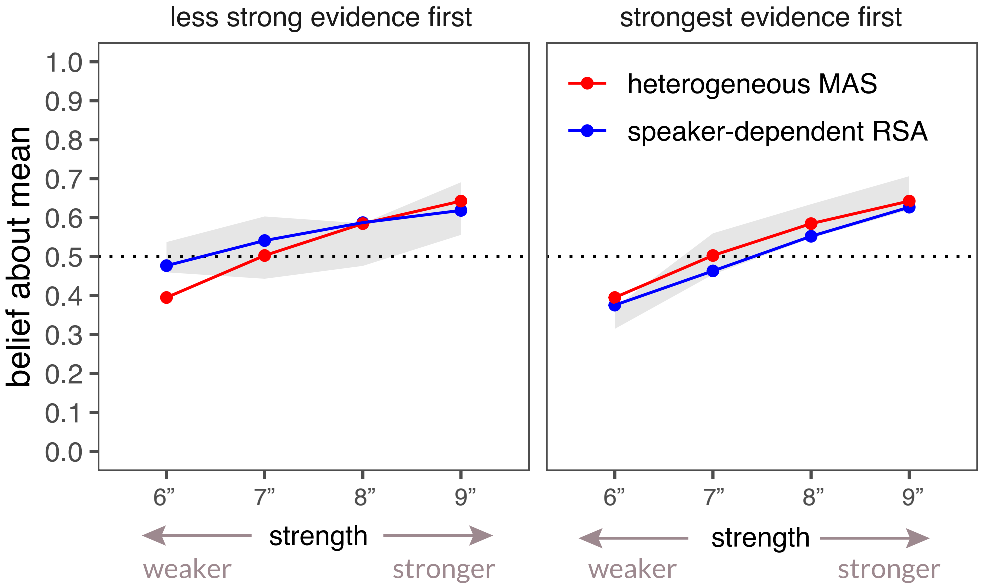

Figure S4: We visualized the posterior predictives for the speaker-dependent RSA model (blue) and heterogeneous MAS model (red). The facets represent which stick was expected to be chosen first in the speaker phase, and the grey region represents the 95% confidence interval of the empirical data.

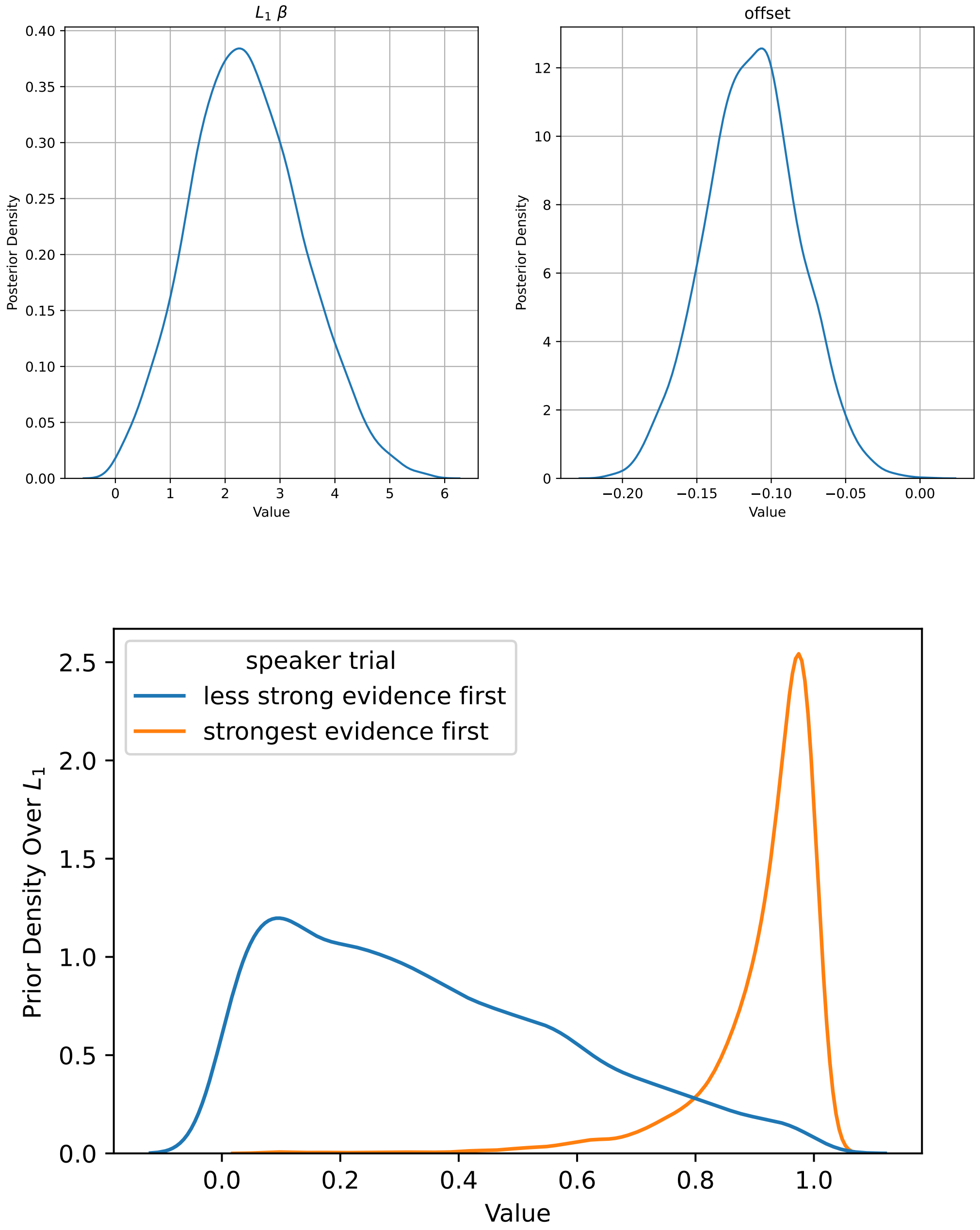

Figure S5: Full Bayesian posteriors for the parameters of the speaker-dependent RSA model. In the top panel, the MAP parameter values are found to be $(\beta^{*}, o^{*}) = (2.26, -0.11)$. The bottom panel shows the posteriors over mixture weights p_z for the different speaker groups. The MAP parameter values for are $p_z = 0.10$ for the less strong evidence group and $p_z = 0.97$ for the strongest evidence group.

group	What was your strategy for selecting sticks as a speaker?
strongest evidence	If I need them to believe more than 5 inch i'd choose the biggest and opposite for below 5 inch — *either choose the longest if I am blue, or the shortest if I am red* — I picked the longest or shortest stick based on what I wanted the judge to believe — *Trying to show the extremes for each argument so the judge thinks the average is more likely to be closer to those* — I think it's best to show the longest/shortest stick you own - to make it appear that they're all very long/short — *i guess it was to show extremes of the sizes of sticks i had, show the smallest on or the tallest one* — my strategy was to create the illusion that the average lenght is bigger in the case I am the blue contestant by showing the longest sticks only, and the same with the red one only showing the shortest. — *Pick the shortest or longest one to bump up or reduce the avearge*
weaker evidence	Selected slightly towards where the first stick suggested — *I actually want to avoid the highest or lowest if I can at first to give the impression that you yourself have picked a more "average" stick.* — Not going too far either way, but just enough to seem less obvious. — *To show a slightly longer or shorter length than the average to try persuade the judge otherwise.* — show some variation to gain trust — *try to keep them guessing*

Table S2: Participants were presented with a free-response text field to explain their reasoning at the end of both phases. Here we provide sample responses from the end of the *speaker* phase, from both participants who expected the *strongest* evidence and those who expected less strong evidence.

group	**How did you reach your decision as a judge?**
strongest evidence	6 is not very much over the average that their trying to prove - which makes me think that al the other sticks are even shorter than that." — *I was thinking that the pink player would choose the shortest stick, whilst the blue would choose the longest* — if 4cm was the shortest stick available then the maximum number of sticks above 5cm would be 4 — *blue showed me a very long stick meaning there would have to be an opposite short stick to average it out. pink however did not show a very short stick suggesting there aren't any.* — the blue would have shown a longer one if it was there — *I assume that blue would be likely to pick the longest possible stick as they have an incentive to make me think the average is above 5in; if they only present a 6in stick, it is likely that the average is under 5in.*
weaker evidence	tried to do a average — *Felt the pink player was bluffing* — thge average of the 2 sticks was shorter than 5 — *Looking at the average of the values I'd been given so far* — seemed similar to how I played it so assumed there were more long ones to come like in my strategy — *the contestant is likely to trick you*

Table S3: Sample responses from the end of the *judge* phase, from both participants who expected the *strongest* evidence and those who expected less strong evidence.
